# Genetic Dissection of Tissue-Specific Apolipoprotein E Function for Hypercholesterolemia and Diet-Induced Obesity

**DOI:** 10.1371/journal.pone.0145102

**Published:** 2015-12-22

**Authors:** Tobias Wagner, Alexander Bartelt, Christian Schlein, Joerg Heeren

**Affiliations:** Department of Biochemistry and Molecular Cell Biology, University Medical Center Hamburg-Eppendorf, Hamburg, Germany; University of Graz, AUSTRIA

## Abstract

ApoE deficiency in mice (*Apoe* ^−/−^) results in severe hypercholesterolemia and atherosclerosis. In diet-induced obesity, *Apoe* ^−/−^ display steatohepatitis but reduced accumulation of triacylglycerides and enhanced insulin sensitivity in white adipose tissue (WAT). Although the vast majority of apoE is expressed by hepatocytes apoE is also abundantly expressed in WAT. As liver and adipose tissue play important roles for metabolism, this study aims to outline functions of both hepatocyte- and adipocyte-derived apoE separately by investigating a novel mouse model of tissue-specific apoE deficiency. Therefore we generated transgenic mice carrying homozygous floxed *Apoe* alleles. Mice lacking apoE either in hepatocytes (*Apoe* ^ΔHep^) or in adipose tissue (*Apoe* ^ΔAT^) were fed experimental diets. *Apoe* ^ΔHep^ exhibited slightly higher body weights, adiposity and liver weights on diabetogenic high fat diet (HFD). Accordingly, hepatic steatosis and markers of inflammation were more pronounced compared to controls. Hypercholesterolemia evoked by lipoprotein remnant accumulation was present in *Apoe* ^ΔHep^ mice fed a Western type diet (WTD). Lipidation of VLDL particles and tissue uptake of VLDL were disturbed in *Apoe* ^ΔHep^ while the plasma clearance rate remained unaltered. *Apoe* ^ΔAT^ did not display any detectable phenotype, neither on HFD nor on WTD. In conclusion, our novel conditional apoE deletion model has proven here the role of hepatocyte apoE for VLDL production and diet-induced dyslipidemia. Specific deletion of apoE in adipocytes cannot reproduce the adipose phenotype of global *Apoe* ^−/−^ mice, suggesting that apoE produced in other cell types than hepatocytes or adipocytes explains the lean and insulin-sensitive phenotype described for *Apoe* ^−/−^ mice.

## Introduction

Overconsumption of high-caloric diets and obesity are major risk factors for abnormal hepatic function in the Western world. In conjunction with obesity, non-alcoholic steatohepatitis (NASH) is associated with insulin resistance and is further characterized by metabolically elicited chronic inflammation of the liver as well as the adipose tissue [1,2]. Both increased lipid production by the liver and the disability of adipose tissue to sequester excess lipids in an efficient and safe fashion lead to accumulation of lipoproteins in plasma and an atherogenic lipoprotein profile [3–6]. Despite the crucial role of obesity and dyslipidemia in the development of the metabolic syndrome, the functional relationship between adipose tissue and hepatic lipoprotein metabolism is not completely understood. Apolipoprotein E (apoE) is a secreted glycoprotein that plays an important part in lipid and lipoprotein metabolism: Being a major component of triglyceride-rich lipoproteins (TRL), it mediates the clearance of postprandial chylomicrons and VLDL remnants by the liver through interaction with LDL receptor (LDLR), LDL receptor related protein 1 (LRP1) and heparan sulphate proteoglycans (HSPG) [7,8]. Additionally, apoE facilitates reverse cholesterol transport and thus supports the maintenance of plasma lipid homeostasis and the removal of atherogenic lipoproteins [9–11]. *Apoe* ^−/−^ mice are characterized by high remnant lipoprotein and low HDL levels, making *Apoe* ^−/−^ mice one of the most commonly used atherosclerosis models [11–14]. Furthermore, according to studies in transgenic mice apoE is also implicated in VLDL assembly and in the regulation of vascular lipolysis [15–18]. *Apoe* ^−/−^ mice exhibit lower HFD-induced weight gain, WAT mass and improved insulin sensitivity while steatohepatitis is more pronounced [18–20]. One major limitation of the aforementioned studies is the usage of globally apoE deficient mice. In particular, plasma apoE is not only synthesised by the liver [21] and macrophages [22,23], but apoE is also highly expressed in adipose tissue and the brain [24–26]. To date, the role of endogenous hepatocyte and adipocyte apoE has predominantly been studied in cultured cells derived from *Apoe* ^−/−^ mice or by tissue transplantation [27].

In the current study we aim to genetically dissect the tissue-specific roles of apolipoprotein E for diet-induced obesity and hypercholesterolemia using a novel conditional transgenic mouse model. Here we show that conditional deletion of hepatocyte apoE using Cre-loxP technology mimics the metabolic features of global *Apoe* ^−/−^ mice with enhanced steatosis. However, hepatic apoE expression has no influence on WAT mass and insulin resistance neither on chow, diabetogenic nor on atherogenic diets. Furthermore, adipocyte-specific apoE ablation did not influence weight gain and inflammation in WAT indicating that apoE from other sources is sufficient to maintain apoE function in adipose tissue.

## Materials and Methods

### Animals and diets

Animals were maintained on a 12 hrs. / 12 hrs. light/dark cycle at 22°C with free access to food and water. All animal procedures were performed with approval from the animal care committee of the University Medical Centre Hamburg-Eppendorf. The tissue-specific disruption of *Apoe* was achieved by use of a Cre/loxP system. Vector construction and targeted knockout strategy was designed together with genOway (Lyon, France). An appropriate targeting vector has been constructed displaying the following features: asymmetric homology arms isogenic with ES cell line (129/Sv), insertion of two *lox*P sites flanking *Apoe* exons 2 to 4, presence of a positive neomycin gene flanked by FRT sites and presence of Diphtheria toxin A negative selection marker. The *Apoe* targeting vector was electroporated into 129Sv ES cells and resistant ES cell clones were isolated and screened by PCR and Southern Blot analysis. Selected ES cell clones were expanded and used for blastocyst injection. Chimeric males were crossed with Flp recombinase expressing delete mice resulting in the generation of Neo-excised, heterozygous *Apoe* floxed mice. These mice were backcrossed with C57BL6/J for at least 10 generations.

To generate a hepatocyte-specific *Apoe* knockout, Cre recombinase was expressed under the control of mouse Albumin promoter (purchased from Jackson, stock number #018961). In a second mouse line, Cre was coupled to mouse Fabp4 gene promoter to achieve adipose-specific *Apoe* depletion (purchased from Jackson, stock number #005069). The following primers were used for detection of floxed alleles: GTGGCCCTGTCCCAAGCACCTCTCT, CCCATGCCTACAATCCAGGGGTAGG and for the detection Alb-Cre: GCACTGATTTCGACCAGGTT, CCCGGCAAAACAGGTAGTTA and aP2-Cre: GCGGTCTGGCAGTAAAAACTATC, GTGAAACAGCATTGCTGTCACTT. Male mice were fed with a diabetogenic high-fat diet (HFD, Bio-Serv F3282, 35% fat), with a control diet (Bio-Serv F4031, 6% fat) or with a western type diet (WTD, ssniff EF R/M acc. TD88137 mod., 21.2% fat, 33.2 sugar, 2.07 mg kg^-1^ Cholesterol) ad libitum single-caged.

### Oral glucose tolerance tests

Oral glucose tolerance tests (OGTT) were performed by gavage of 1 g/kg in 200 μL. Blood glucose levels were monitored at the beginning and 15, 30, 60, 90 and 120 minutes after gavage by use of AccuChekAviva sticks (Roche).

### Tissue harvesting

After 16 weeks of feeding, mice were euthanized, blood samples were taken and perfused with PBS containing 50 U/ml heparin. Tissue were dissected, snap-frozen in liquid nitrogen and stored at -80°C for subsequent analysis.

### Plasma analysis

Blood was collected in Eppendorf tubes with EDTA and placed on ice until separation by centrifugation. After centrifugation, plasma samples were collected and stored at -80°C for subsequent analysis. Plasma cholesterol and triacylglycerides were measured by commercial kits (Roche). ApoE plasma levels were determined by ELISA as described before [28]. ALT plasma levels were measured by Cobas Mira analyser (Roche) according to the manufacturer’s manual.

### Plasma protein profiles

Plasma proteins of pooled plasma samples were separated using S6-superose sizing columns on AKTA FPLC (GE Healthcare).

### VLDL production

After 4 weeks of chow or WTD, mice were fasted for 5 hours, followed by an adapted intravenous injection of Tyloxapol (Sigma Aldrich) equivalent to 0.5 g kg^-1^ body weight. Aliquots of tail vein blood were taken at the beginning and 30, 60 and 120 min after injection for plasma TG determination.

### Liver lipids

100 mg of snap-frozen hepatic tissue were homogenised by 10 μL mg^-1^ lysis buffer (80 mM NaCl, 2 mM CaCl_2_, 1% Triton X100 and 50 mM Tris-HCl at pH 8). Triglyceride and cholesterol concentrations in the homogenates were determined as described above. Measurements were normalized on hepatic protein content. For that, protein lysates were prepared by RIPA buffer (50 mM NaCl, 50 mM NaF, 20 mM Tris-HCl, 10 mM Na_2_H_2_P_2_O_7_, 5 mM EDTA, 1 mM Na_3_VO_4_, 1% Nonidet-P40, 1% SDS and protease inhibitor (Roche) and TissueLyser (Qiagen) and protein concentrations were measured by Lowry method.

### Turnover of apoE-free VLDL particles

ApoE knockout mice were fasted for 5 hours and blood samples were harvested by cardiac puncture under anaesthesia. VLDL particles were isolated by sequential ultracentrifugation. VLDL particles were labelled by ^125^I-tyramine cellubiose (^125^I-TC). For turnover analysis, mice were fasted for 5 hours before tracer injection in a tail vein. Afterwards, blood samples were taken at 2, 5, 10, 20 and 30 minutes after injection. After 30 minutes, animals were euthanized and the livers were perfused with PBS. After perfusion with PBS-heparin, the liver, the heart, epididymal, subcutaneous and subscapular fat pads as well as kidney, spleen and muscle were harvested, homogenized and assayed for ^125^I radioactivity. For analysis, the counts per minute (cpm) were normalized by sample weight.

### RNA extraction and real-time PCR

Total RNA was extracted from tissues using TissueLyser (Qiagen) and Trizol (Invitrogen) and purified by NucleoSpin RNAII kit (Macherey and Nagel) according to the manufacturer’s advices. RNA concentration was determined with a PeqLab NanoDrop 1000. First strand cDNA was reverse transcribed from 1 μg of total RNA by High-Capacity cDNA Archive Kit (Applied Biosystems) according to the manufacturer’s instructions. Real-time RT-PCR reactions were performed by use of assay-on-demand primer sets supplied by Applied Biosystems. Reactions were conducted using TaqMan Gene Expression Analysis (Applied Biosystems) and normalized on housekeeper mRNA (mTbp) by ΔΔCt method as described previously [29].

### Western blotting

Snap-frozen samples were lysed by TissueLyser (Qiagen) in RIPA buffer (supplemented with complete mini protease inhibitor cocktail [Roche]), Na_3_VO_4_ (50 mM) and 0.1%-SDS. Proteins were separated on NuPAGE Bis-Tris polyacrylamide gels (Invitrogen). ApoE was detected by commercial apoE antibody (Santa Cruz).

### Histology

Liver pieces were fixed in 3.5% Para-formaldehyde solution and paraffin-embedded. 10 μm slices (Leica Microtom) were stained with Haematoxylin and Eosin and steatosis was assessed optically by evaluation of the tissue structure and the frequency of lipid droplets within the hepatocytes. Pictures from histological sections of EpiWAT (>10 independent pictures per mouse, with n = 3 per group) were used for the calculation of adipocyte size distribution using manual detection by ImageJ.

### Statistical analysis

Data are expressed as means ± SEM. Student’s t test was used for comparison between groups, a p-value <0.05 was assumed to indicate significant differences.

## Results

### Conditional apoE deletion in hepatocytes or adipocytes

**Fig 1:**
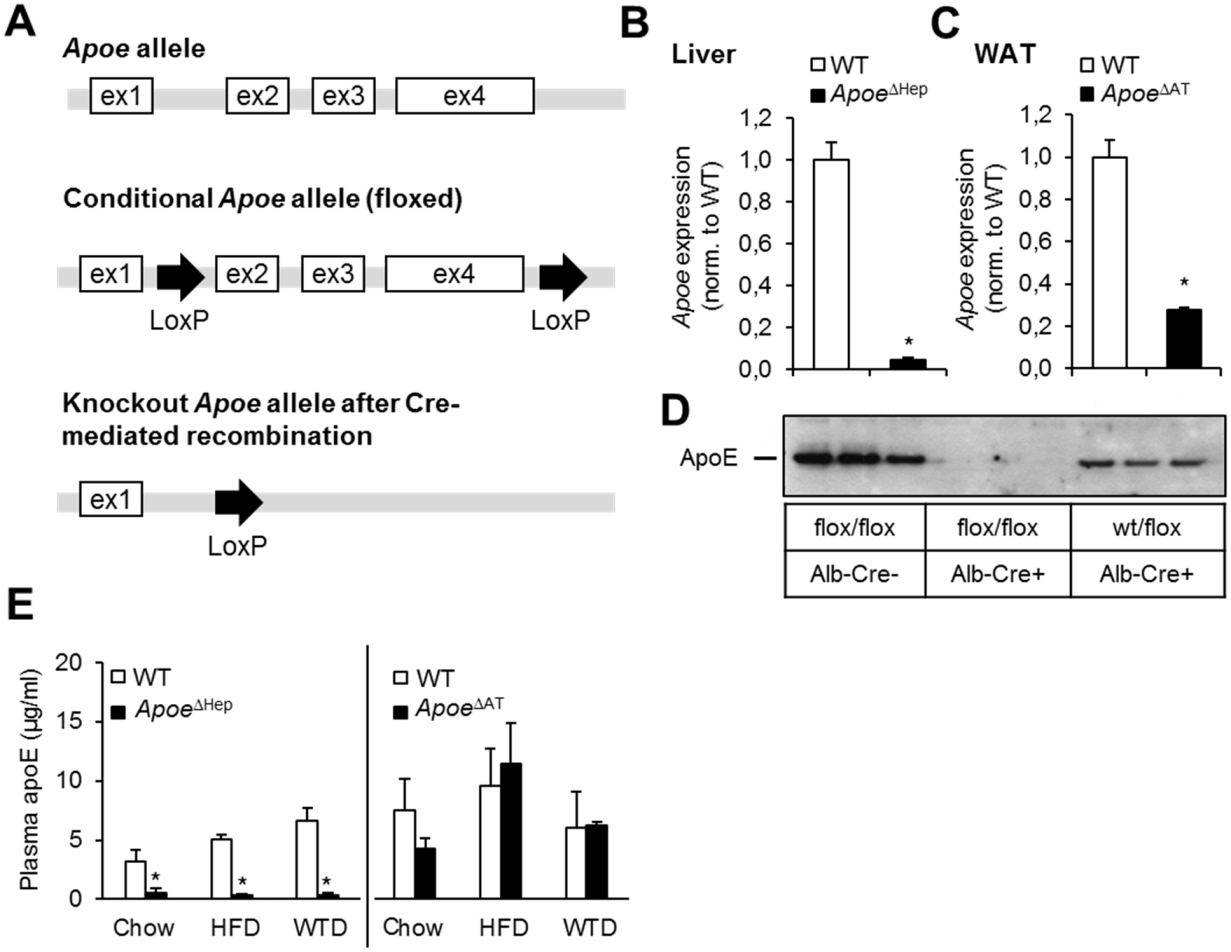
Conditional deletion of apoE in mice using Cre/loxP system. (A) Scheme of the *Apoe* allelels flanked by loxP sites. (B,C) *Apoe* gene expression level of generated knockout models of either (B) liver of *Apoe* ^ΔHep^ mice (n≥8) and (C) adipose tissue of *Apoe* ^ΔAT^ mice (n≥6). (D) Total apoE protein amount of liver lysates of Cre- and Cre+ mice crossed with either wt/Apoe flox or Apoe flox/flox mice as indicated. ApoE protein was detected at 34kDa by Western blot (n≥3). (E) ApoE plasma levels of *Apoe* ^ΔHep^ and *Apoe* ^ΔAT^ mice after feeding chow, a diabetogenic high fat diet (HFD or a cholesterol-containing Western type diet (WTD). Apoe^ΔHep^ and respective controls on chow (n≥5), HFD (n≥8) and WTD (n≥6); Apoe^ΔAT^ and respective controls on chow (n≥7), HFD (n≥8) and WTD (n≥3). WT: Wild-type. WAT: white adipose tissue. Mean values ± s.e.m. Statistical analysis with *Students T-Test*. *: p<0.05.

In order to investigate the specific role of apoE produced by hepatocytes or adipocytes, we generated a conditional transgenic mouse model using Cre-loxP technology, in which critical regions of the *Apoe* gene can be deleted in a cell type-specific manner (Fig 1A). The Cre mediated recombination leads to the dissection of exons 2 to 4, the region that has been chosen for the generation of the global *Apoe* ^*-/-*^ mice [12]. In order to delete apoE in hepatocytes we crossed a transgenic line under control of the *Alb* promoter (*Apoe* ^ΔHep^). For deletion in adipose tissue, recombination was initiated by transgenic expression of *Fabp4*-Cre (*Apoe* ^ΔAT^). As controls, we used littermates carrying homozygous floxed *Apoe* alleles but no Cre recombinase (WT).

As expected, *Apoe* expression was diminished in the livers of *Apoe* ^ΔHep^ mice (Fig 1B) and in WAT of *Apoe* ^ΔAT^ mice (Fig 1C). Near-total depletion of hepatic apoE in homozygous floxed *Apoe* ^ΔHep^ was further verified by Western blotting of liver extracts (Fig 1D). In order to investigate the significance of hepatocyte- and adipocyte-specific apoE deletion on plasma apoE levels, we performed feeding studies using a diabetogenic high-fat diet (HFD), a cholesterol-enriched Western type diet (WTD) or a standard rodent chow control diet for 16 weeks beginning at 4 weeks of age. The reduction in hepatic apoE in *Apoe* ^ΔHep^ mice under these different dietary regimens translated into diminished levels of apoE in plasma whereas in *Apoe* ^ΔAT^ mice plasma levels were unchanged compared to WT controls (Fig 1E). These data confirm that the pool of circulating plasma apoE is mainly constituted by hepatic production. In summary, this novel model allows for studying the tissue-specific actions of apoE.

### Effect of dietary regimen on weight gain and adiposity in hepatocyte- and adipocyte-specific apoE deficient mice

**Fig 2:**
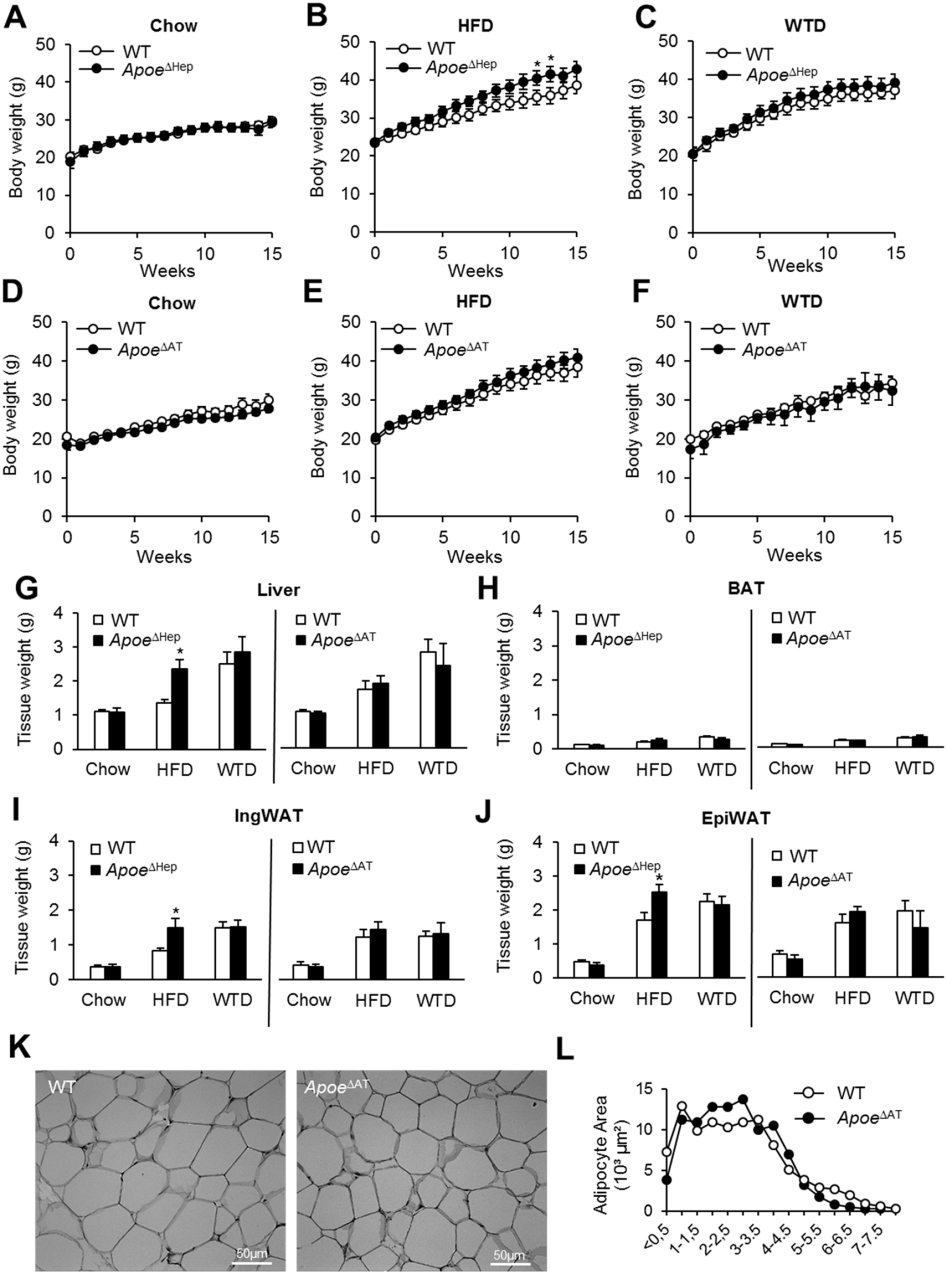
Effect of tissue-specific deletion of apoE on Western and HFD feeding. (A-C) Body weight curves of *Apoe* ^ΔHep^ or (D-F) *Apoe* ^ΔAT^ mice fed either chow (A, D), HFD (B, E) or WTD (C,F). Tissue weights of (G) liver, (H) interscapular BAT, (I) IngWAT and (J) EpiWAT after 16 weeks on each diet (chow, HFD, WTD) of Apoe^ΔHep^ or Apoe^ΔAT^ mice. Apoe^ΔHep^ and respective controls on chow (n≥5), HFD (n≥7) and WTD (n≥6); Apoe^ΔAT^ and respective controls on chow (n≥7), HFD (n≥9) and WTD (n≥3). (K) Representative pictures from EpiWAT sections of HFD fed mice from Apoe^ΔAT^ and respective controls. (L) Calculation of adipocyte size distribution using ImageJ (n≥3). WT: Wild type mice. HFD: high fat diet. WTD: Western type diet. EpiWAT: epididymal white adipose tissue. IngWAT: inguinal white adipose tissue. iBAT: interscapular brown adipose tissue. Mean values ± s.e.m. Statistical analysis with *Students T-Test*. *: p<0.05.

ApoE has been implicated in the response to dietary cholesterol intake and diet-induced obesity and hence we studied the phenotypic consequences of tissue-specific apoE deletion under the aforementioned dietary regimens. *Apoe* ^ΔHep^ mice displayed a tendency towards higher body weights on HFD (about 10%) while on either chow or WTD weight gain was similar (Fig 2A–2C). Differences in weight gain of *Apoe* ^ΔHep^ mice were not based on altered daily food intake (S1 Fig). However, *Apoe* ^ΔAT^ mice did not show genotype-specific alterations in weight gain (Fig 2D–2F).

Compared to WT controls, we observed an increase in liver mass of *Apoe* ^ΔHep^ mice fed HFD (Fig 2G). The weights of interscapular brown adipose tissue (BAT) were unchanged between genotypes (Fig 2H). In addition and according to the increased body weights, white adipose tissue fat pad mass from inguinal (IngWAT) and epididymal (EpiWAT) depots were significantly higher in Apoe^ΔHep^ mice on HFD compared to respective HFD-fed WT controls (Fig 2I and 2J). The loss of apoE expression in adipose tissue did not influence liver, BAT and WAT depot mass (Fig 2G, 2H, 2I and 2J). Also the histological appearance of adipose tissue, adipocyte size distribution in epiWAT (Fig 2K and 2L) and oral glucose tolerance (S2 Fig) were similar between controls and Apoe^ΔAT^. These results demonstrate that neither *Apoe* ^ΔHep^ nor *Apoe* ^ΔAT^ display a similar protection from WTD- or HFD-induced weight gain and adiposity as it has been described for global *Apoe* ^−/−^ mice [18–20].

### *Apoe* ^ΔHep^ mice display increased hepatic cholesterol content

**Fig 3:**
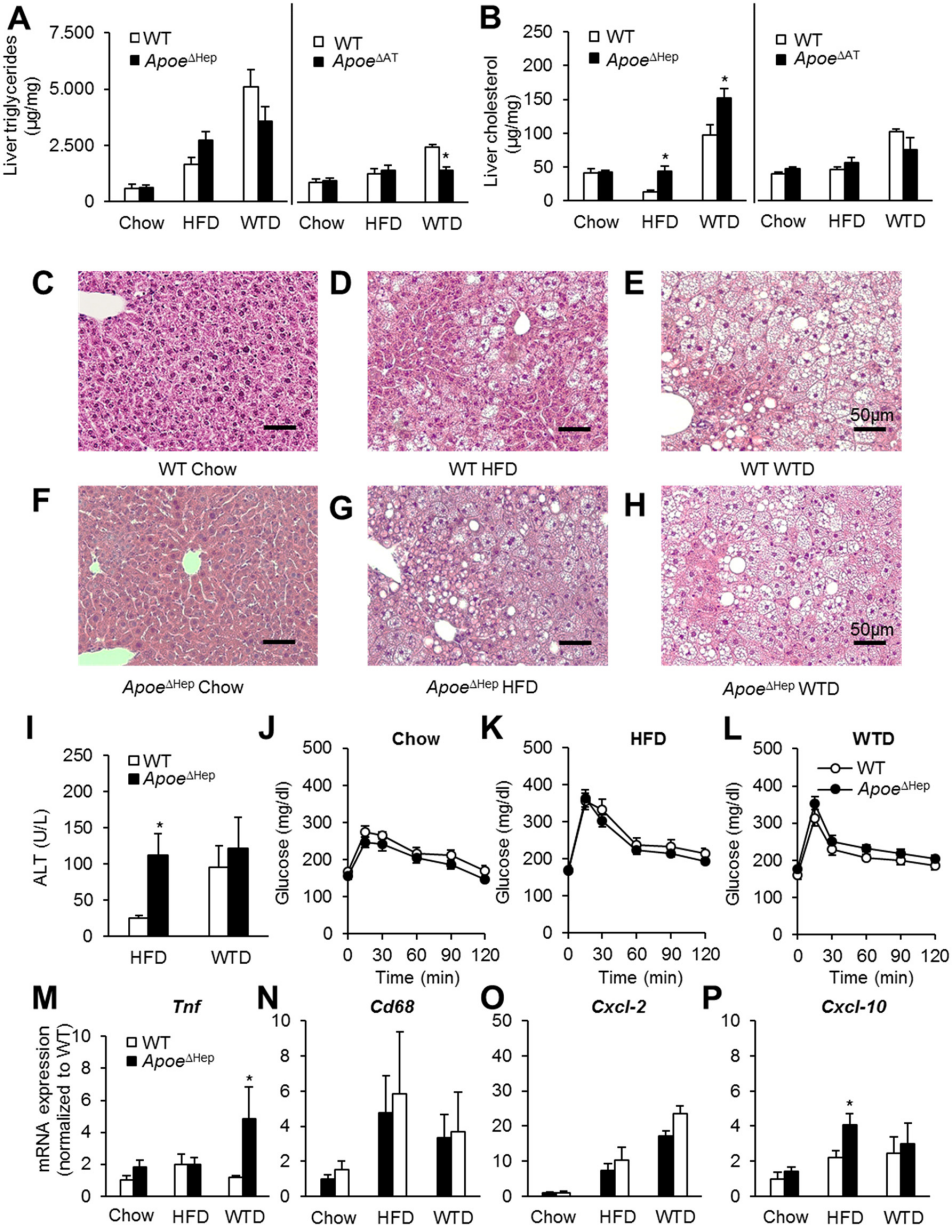
Hepatocyte apoE modulates liver steatosis and hepatic inflammation. (A) Total liver triglyceride and (B) cholesterol measurement of *Apoe* ^ΔHep^ and *Apoe* ^ΔAT^ mice 16 weeks after feeding a chow, HFD or WTD diet. Apoe^ΔHep^ and respective controls on chow (n≥5), HFD (n≥5) and WTD (n≥6); Apoe^ΔAT^ and respective controls on chow (n≥5), HFD (n≥6) and WTD (n≥3). Haematoxylin-Eosin stained liver sections of (C-E) WT controls or (F-H) *Apoe* ^ΔHep^ on (C,F) chow, (D,G) HFD or(E,H) WTD. Scale bar: 50μm. (I) Plasma ALT activity of control and *Apoe* ^ΔHep^ mice on HFD or WTD. (J-L) Oral glucose tolerance test of *Apoe* ^ΔHep^ mice on either (J) chow, (K) HFD or (L) WTD. (M-P) Liver gene expression of (M) *Tnf*, (N) *Cd68*, (O) *Cxcl-2* and (P) *Cxcl-10*. I, M-P: Apoe^ΔHep^ and respective controls on chow (n≥4), HFD (n≥5) and WTD (n≥4). J, K, L: Apoe^ΔHep^ and respective controls on chow (n≥5), HFD (n≥8) and WTD (n≥6). WT: Wild type mice. HFD: high fat diet. WTD: Western type diet. Mean values ± s.e.m. Statistical analysis with *Students T-Test*. *: p<0.05.

As apoE has been described to influence hepatic lipid metabolism [15,20,30,31], we investigated the liver phenotype in *Apoe* ^ΔHep^ and *Apoe* ^ΔAT^ mice. To quantify the hepatic lipid content, hepatic triglyceride and cholesterol levels were measured under different dietary conditions (Fig 3A and 3B). Hepatic triglyceride levels were increased by both HFD and WTD but displayed unaltered upon deficiency of hepatic apoE (Fig 3A). We found elevated levels of cholesterol in *Apoe* ^ΔHep^ both on HFD and WTD (Fig 3B). The increase in liver mass in in *Apoe* ^ΔHep^ mice fed HFD was also associated with histological signs of steatosis (Fig 3C–3H).

In order to determine liver damage, alanine aminotransferase (ALT) plasma activity was determined in *Apoe* ^ΔHep^ and WT controls on HFD and WTD. On HFD, ALT activity was 4-fold higher in mice lacking hepatocyte apoE while ALT activity reached comparable levels on WTD (Fig 3I). WT controls mice also displayed enhanced ALT levels on WTD. Therefore it is conceivable that apoE-dependent effects are masked under these dietary conditions (Fig 3I). However, differences in hepatic steatosis did not correlate with any changes in oral glucose tolerance (Fig 3J–3L). Markers of hepatic inflammation remained unchanged largely between genotypes except enhanced *Tnf* expression on WTD (Fig 3M–3P). Thus, in the context of HFD feeding *Apoe* ^ΔHep^ resemble to a certain degree the hepatic phenotype of global apoE deficiency [20].

### Dyslipidemia in *Apoe* ^ΔHep^ is dependent on dietary cholesterol

**Fig 4:**
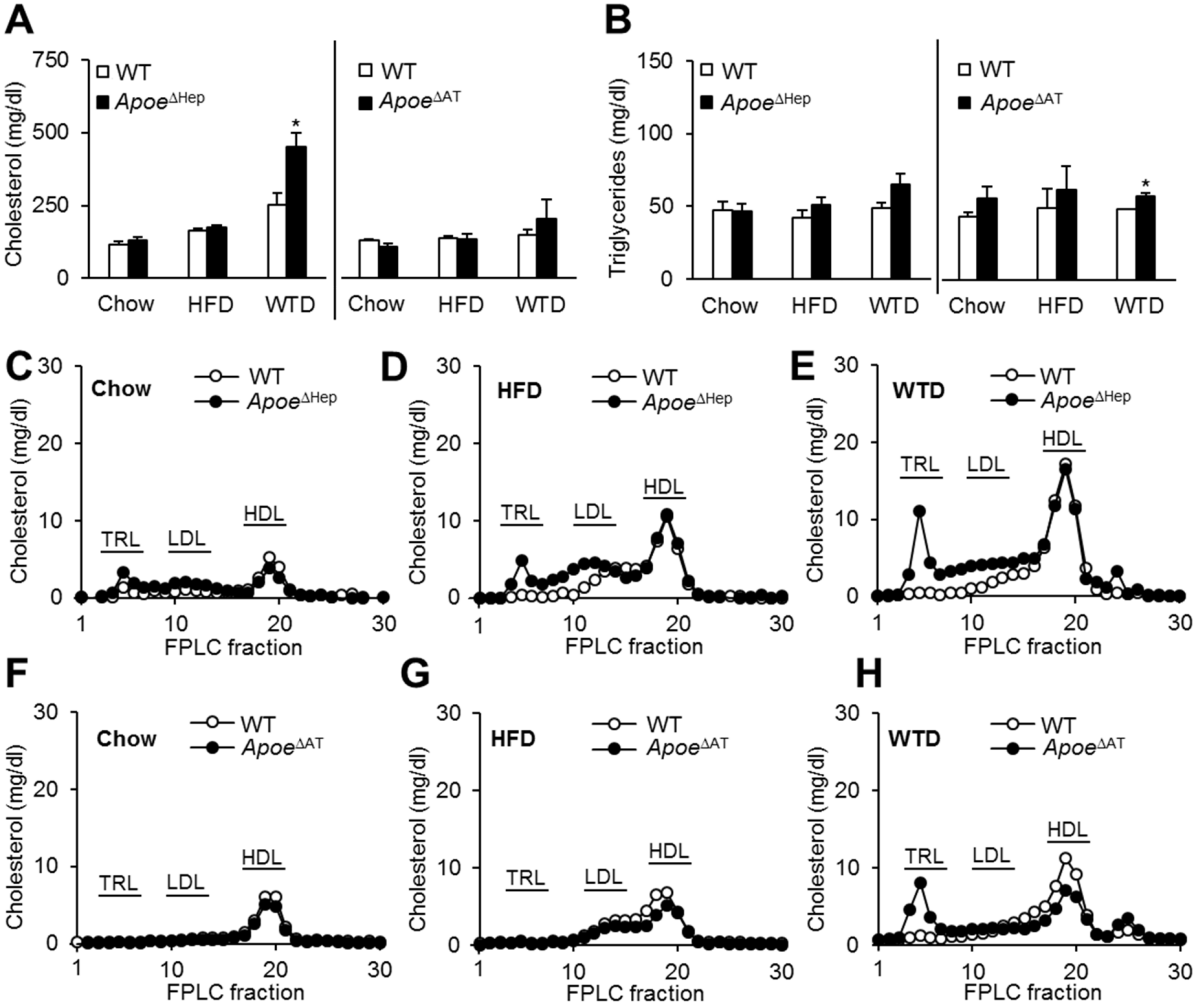
Loss of hepatic apoE leads to dyslipidemia on Western type diet. (A) Total plasma cholesterol (B) or triglyceride level in *Apoe* ^ΔHep^ and *Apoe* ^ΔAT^ mice on chow, HFD and WTD. Apoe^ΔHep^ and respective controls on chow (n≥8), HFD (n≥6) and WTD (n≥6). Apoe^ΔAT^ and respective controls on chow (n≥7), HFD (n≥8) and WTD (n≥3). (C-E) FPLC profiles of pooled samples for cholesterol in *Apoe* ^ΔHep^ mice or (F-H) in *Apoe* ^ΔAT^ mice on (C,F) chow, (D,G) HFD or(E,H) WTD. WT: Wild type mice. HFD: high fat diet. WTD: Western type diet. Mean values ± s.e.m. Statistical analysis with *Students T-Test.* *: p<0.05.

Plasma cholesterol levels were similar between genotypes on either chow or a HFD (Fig 4A). Compared to WT controls, however, *Apoe* ^ΔHep^ mice displayed increased plasma cholesterol when fed a WTD. No differences in plasma triglycerides were found (Fig 4B). *Apoe* ^ΔAT^ mice displayed plasma cholesterol levels comparable to WT mice on each diet whereas slightly higher plasma triglyceride levels could be detected in WTD fed Apoe^ΔAT^ mice (Fig 4A and 4B). Lipoprotein profiling by FPLC revealed increased cholesterol in the TRL and remnant fractions of *Apoe* ^ΔHep^ mice, being most pronounced on WTD (Fig 4C–4E). Interestingly, in *Apoe* ^ΔAT^ a surplus of cholesterol-rich TRL remnants was limited to WTD (Fig 4F–4H) accompanied by reduced HDL cholesterol load resulting in unchanged total plasma cholesterol levels. The FPLC triglyceride profiles did not show any significant differences (data not shown). Taken together, lack of hepatic apoE is in particular important for plasma cholesterol homeostasis under conditions of increased dietary cholesterol intake.

### VLDL production and delivery are dependent on hepatocyte apoE

**Fig 5:**
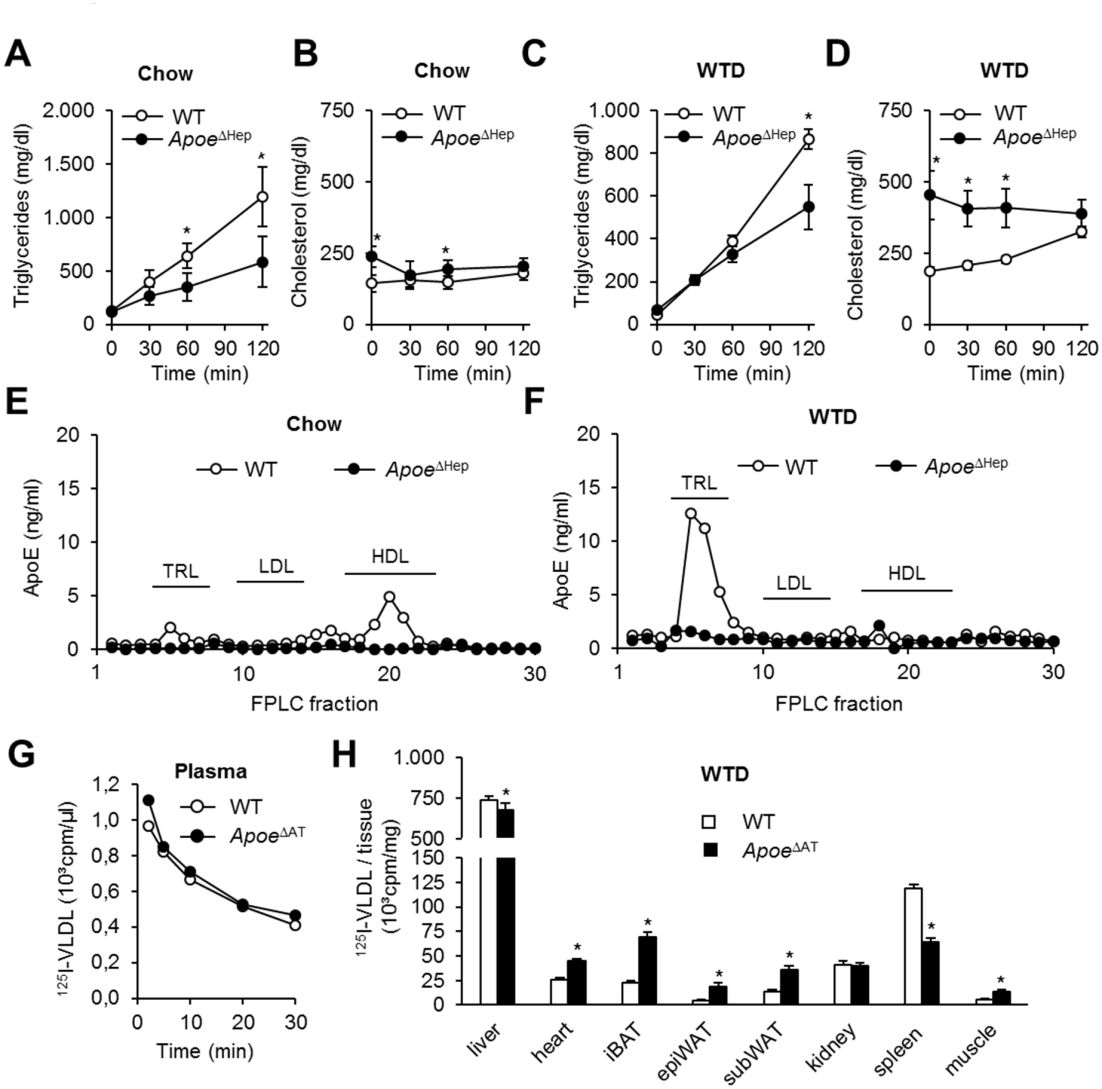
Hepatic apoE regulates VLDL production and lipoprotein uptake. (A-D) VLDL production after tylaxopol injection in *Apoe* ^ΔHep^ mice on (A,B) chow or (C,D) Western type diet. Apoe^ΔHep^ and respective controls on chow (n≥8) and WTD (n≥7). (E,F) Plasma apoE profile by FPLC and apoE-ELISA on (E) chow or (F) Western type diet. (G,H) ApoE-free ^125^I-VLDL turnover in *Apoe* ^ΔHep^ mice with (G) plasma decay and (H) organ distribution (n≥5). WT: Wild type mice. WTD: Western type diet. Mean values ± s.e.m. Statistical analysis with *Students T-Test.* *: p<0.05.

The dietary-induced changes in the lipoprotein profiles observed in *Apoe* ^ΔHep^ mice suggest a role of hepatocyte apoE for VLDL metabolism. As determined by the injection of Tyloxapol, secretion of VLDL triglycerides into plasma is lower in *Apoe* ^ΔHep^ mice both on chow and WTD (Fig 5A and 5C). VLDL cholesterol levels remained unchanged suggesting impaired triglyceride incorporation during hepatic VLDL maturation (Fig 5B and 5D). ApoE content determined in FPLC fractions of pooled plasma samples indicated that under chow conditions apoE is primarily detectable in HDL fractions. In WT mice, feeding a WTD led to enhanced apoE concentration in the TRL fractions while *Apoe* ^ΔHep^ animals showed diminished apoE concentrations on both chow and WTD in these fractions (Fig 5E and 5F).

ApoE is known to interact with different lipoprotein receptors, thus facilitating lipoprotein remnant clearance. Therefore we analyzed whether hepatocyte apoE deficiency might disturb intravascular VLDL delivery to the liver and peripheral tissues. ApoE-free VLDL particles isolated from *Apoe* ^−/−^ mice were labeled by ^125^I-tyramine cellubiose and *in vivo* VLDL turnover experiments were performed (Fig 5G and 5H). While plasma half-life was similar between genotypes (Fig 5G), the organ-specific VLDL distribution was shifted from the liver to other peripheral organs, including BAT [32], indicating that remnant delivery to the liver is attenuated in *Apoe* ^ΔHep^. In conclusion, hepatocyte apoE is both necessary for VLDL lipidation as well as lipoprotein remnant clearance by the liver.

## Discussion

In the current study we established a novel mouse model for conditional apoE deficiency to investigate the role of adipocyte and hepatocyte apoE for hypercholesterolemia and diet-induced obesity. While the *Apoe* ^−/−^ mice is a standard model for hypercholesterolemia and apoE has been implicated in body weight control, genetic evidence for tissue specific actions of apoE is scarce. Here we find that apoE produced by hepatocytes is important to maintain plasma lipid homeostasis, in particular when cholesterol is supplemented in the diet.

Since the first characterization of *Apoe* ^−/−^ mice [12,14], the mouse model has been intensively studied as a preclinical model for the development and treatment of atherosclerosis. *Apoe* ^−/−^ mice have been shown to display several key features of this disease, making this models one of the most frequently used in lipid metabolism research. In these mice, an impaired clearance of chylomicron and VLDL remnants results in a pathological increase of plasma cholesterol even on non-cholesterol diet. Interestingly, *Apoe* ^−/−^ mice are also characterized by enhanced hepatic steatosis as well as improved insulin sensitivity [18,20]. Yet whether this is related to the dyslipidemia or a cell-type specific effect of the lack of apoE remains unclear. Here we specify the function of both hepatocyte- and adipose tissue-derived apoE by using genetically targeted hepatocyte and adipose tissue deficiency of apoE.

First, we investigated adipose tissue-targeted deficiency of apoE by using conditional knockouts generated via the Cre/loxP system. Our study provides evidence that adipocyte apoE neither influences total plasma cholesterol nor triglyceride levels or total plasma apoE protein concentrations. This is in line with a recently published study showing that *Apoe* ^−/−^ adipose tissue transplantations to WT mice does not influence apoE plasma level [33]. Interestingly, we found a HDL-to-TRL cholesterol shift in the FPLC profile, limited to the cholesterol-enriched diet-fed mice. Furthermore, apoE is thought to influence adipocyte differentiation [18–20], which is highlighted by smaller adipocyte size in *Apoe* ^−/−^ mice. These putative discrepancies, however, can be largely explained by the persistently high plasma apoE level in the Apoe^ΔAT^. Although *Fabp4-Cre* has been used in many studies to specifically delete floxed genes in adipocytes [34], in recent years it become clear that *Fabp4-Cre* expression is not restricted to adipocytes but can also be induced in macrophages and endothelial cells [35]. Macrophage apoE has been shown to alleviate hyperlipidemia in Apoe^−/−^ mice [22,23]. In addition, the compensation might not be caused by macrophages alone and can also be dependent on the plasma level or other cell types not affected by the used Cre-model. It will be therefore necessary to investigate the adipose tissue-resident macrophages as a source of apoE in a more specific model e.g. using *LysM-Cre*.

The extent of body weight gain on HFD in wild type mice is usually correlated to chronic low grade inflammation and insulin resistance of the adipose tissue [36]. In line, as *Apoe* ^−/−^ mice are protected from HFD-induced obesity [20], they display less hypertrophy of adipose tissue on HFD, lower inflammation levels and increased insulin sensitivity [18]. Neither the reduced fat accumulation nor the influence on inflammation state was observed in the current study in mice lacking apoE in adipose tissue. In line, organ adipose tissue weights or the mean adipocyte size did not differ between these genotypes. Our findings do not exclude a functional role of apoE in the adipose tissues in adipocyte differentiation, lipid uptake and inflammation, but the data suggest that this function of apoE does not follow conclusively adipocyte-derived apoE amount. Ideally, in future the role of secreted apoE versus a non-secreted, endogenous function could be addressed by expressing apoE mutants that are not secreted in cellular systems for adipocytes or other cells and compare the effect of these mutants on adipocyte differentiation or function. Altogether, lack of adipocyte-derived apoE does not influence obesity and associated metabolic disturbances in our conditional deficiency model.

In order to investigate the effect of hepatic apoE we generated the *Apoe* ^ΔHep^ mouse by crossing our floxed *Apoe* mice with Albumin Cre mice that results in deletion of loxP-flanked genes in hepatocytes. Our study underlines the importance of hepatocyte apoE in hepatic lipid metabolism, indicated by the suppression of diet-dependent inflammation and steatosis during high-fat, high-cholesterol intake by regulating VLDL assembly. The effect on VLDL lipidation was independent of the diet, demonstrating that predominantly intracellular hepatocyte apoE but not the amount of exogenous lipids regulates the extent of triglyceride incorporation into VLDL. Interestingly, in *Apoe* ^ΔHep^ mice, observable hypercholesterolemia was limited to the presence of dietary cholesterol, indicating that under basal lipid supply hepatocyte apoE is not required for appropriate clearance of lipoproteins, but is particularly important in the context of dietary cholesterol excess.

Nutrient stress caused by HFD feeding also causes hepatic steatosis and inflammation [1]. Mice deficient in hepatocyte apoE exhibited an impaired lipid balance in the liver that is in line with global apoE deficiency [30]. Our metabolic studies show that hepatocyte apoE is a relevant regulator of VLDL secretion by controlling triglyceride incorporation during VLDL lipidation. The impaired VLDL assembly and hence the total lipid secretion leads to increased amounts of cholesterol stored in the liver, which is linked to steatosis and inflammation. In summary, our data indicate that hepatocyte apoE is necessary for an efficacious protection from inflammatory lipid accumulation in the liver under conditions of high-fat and high-cholesterol diets.

Interestingly, hepatic uptake of apoE-free VLDL is slightly altered in *Apoe* ^ΔHep^ mice and an increased amount of VLDL delivery to adipose tissues, heart and skeletal muscle can be observed. How TRL distribution to peripheral organs is mechanistically affected by hepatocyte-specific apoE deficiency remains ambiguous. The increased uptake of VLDL in the periphery of *Apoe* ^ΔHep^ mice is accompanied by higher fat mass on HFD while food intake remained unchanged. This is different to the phenotype of *Apoe* ^−/−^ mice, which are known to have smaller fat depots [18,20,27]. Furthermore, due to the smaller fat depots, *Apoe* ^−/−^ mice display improved glucose tolerance, which was not observed either in Apoe^ΔHep^ or Apoe^ΔAT^ mice. Thus, apoE from other might be involved in the regulation of energy uptake and energy expenditure in different adipose tissue depots.

## Conclusion

Depletion of hepatocyte apoE seems to be sufficient to enhance hepatic steatosis and inflammation in context of high cholesterol and high caloric food intake. On the other hand, reduced content of apoE directs VLDL particles to adipose tissue and also heart and skeletal muscle shuffling energy for storage and combustion into the periphery. Based on the current study showing the successful generation of an apoE-floxed model, future studies will help to further define the tissue-specific function of apoE for lipid delivery, atherosclerosis, bone growth, energy homeostasis as well as cognitive function.

## Supporting Information

S1 Fig Effect of tissue-specific Apoe deletion on food intake. (TIF)

S2 Fig Effect of adipose tissue-specific deletion of apoE on oral glucose tolerance. (TIF)
