# Supplementary figures and images for: Genetic Dissection of Tissue-Specific Apolipoprotein E Function for Hypercholesterolemia and Diet-Induced Obesity
Source: PLoS One. 2015 Dec 22;10(12):e0145102. doi: 10.1371/journal.pone.0145102 (PMC4687855; doi:10.1371/journal.pone.0145102)

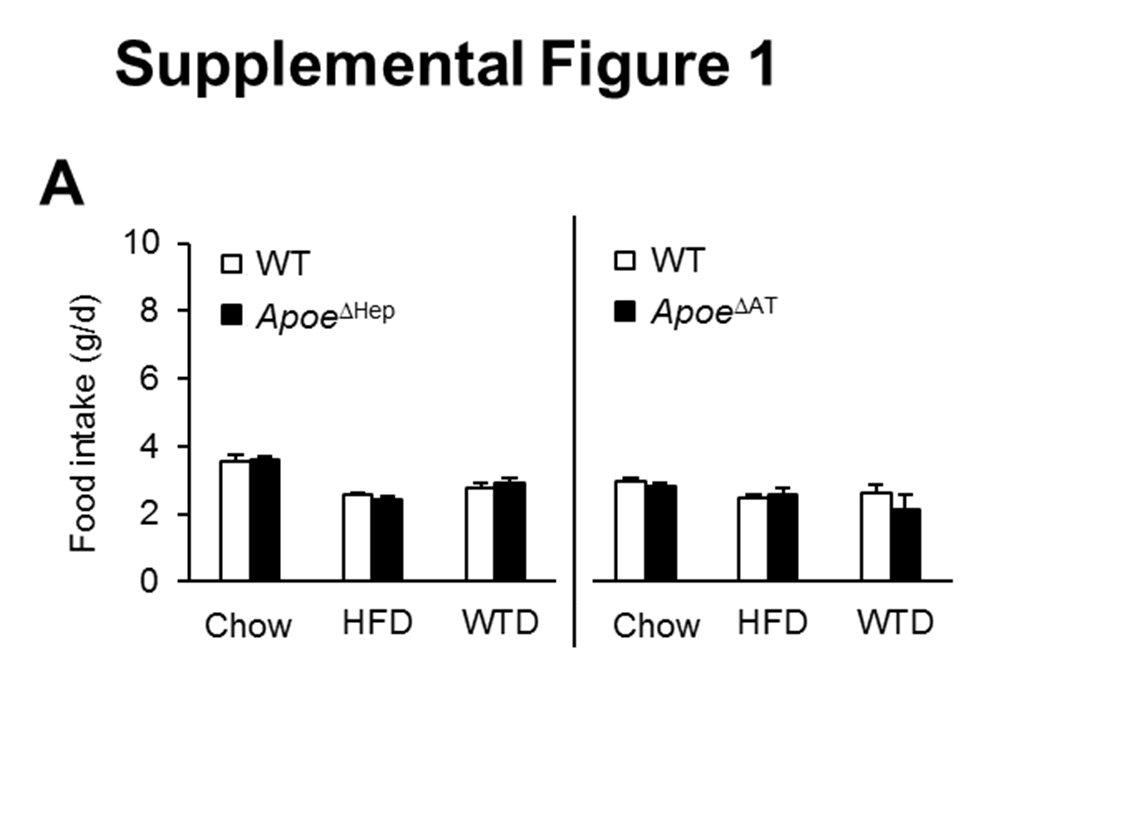

Supplement: S1 Fig — (TIF) [file pone.0145102.s001.TIF]

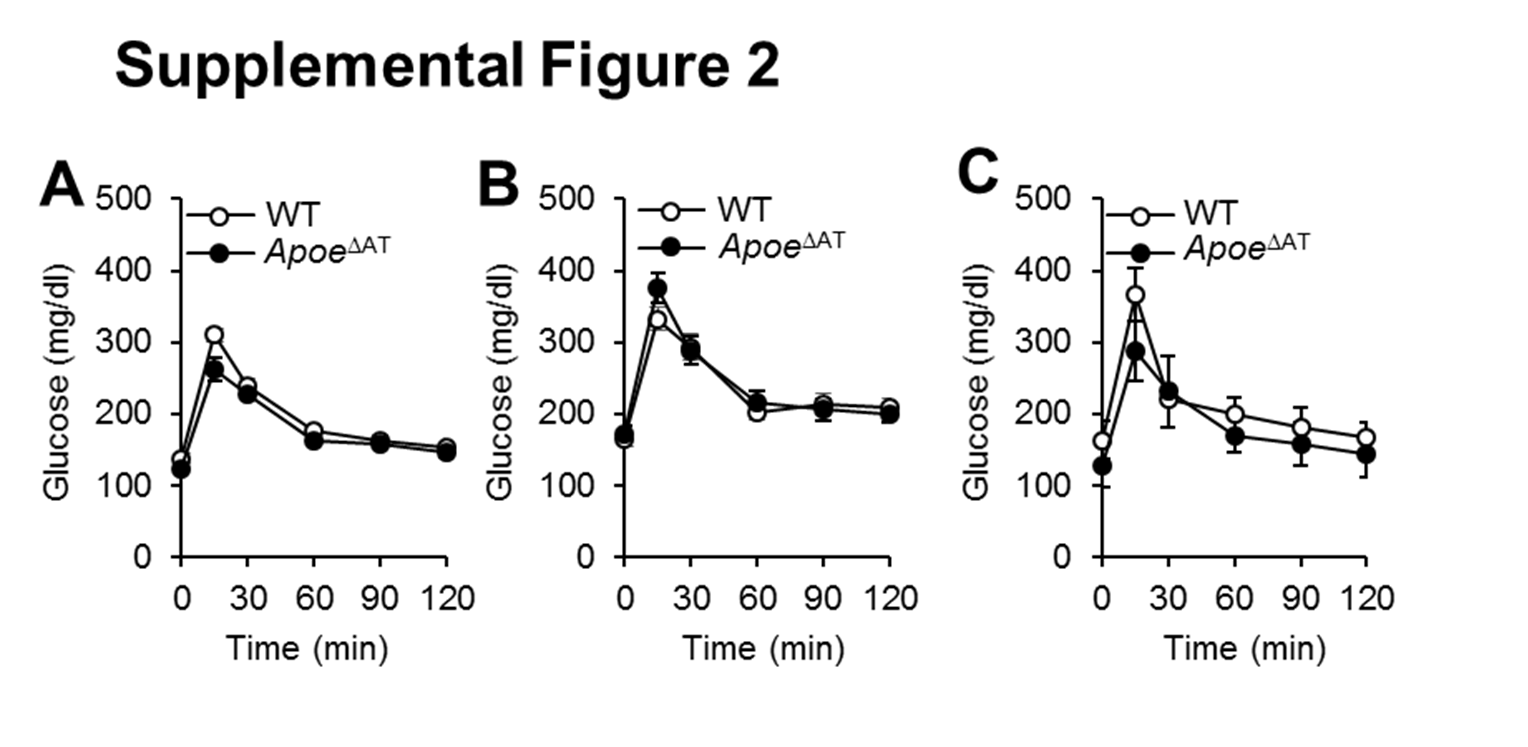

Supplement: S2 Fig — (TIF) [file pone.0145102.s002.TIF]
